# PIV-MyoMonitor: an accessible particle image velocimetry-based software tool for advanced contractility assessment of cardiac organoids

**DOI:** 10.3389/fbioe.2024.1367141

**Published:** 2024-03-12

**Authors:** Hoyeon Lee, Boyoung Kim, Jiyue Yun, Jinseung Bae, Sungsu Park, Junseok Jeon, Hye Ryoun Jang, Jaecheol Lee, Soah Lee

**Affiliations:** ^1^ Department of Biopharmaceutical Convergence, Sungkyunkwan University, Suwon, Republic of Korea; ^2^ School of Electronic and Electrical Engineering, Sungkyunkwan University, Suwon, Republic of Korea; ^3^ School of Mechanical Engineering, Sungkyunkwan University, Suwon, Republic of Korea; ^4^ Division of Nephrology, Department of Medicine, Cell and Gene Therapy Institute, Samsung Medical Center, Sungkyunkwan University School of Medicine, Seoul, Republic of Korea; ^5^ School of Pharmacy, Sungkyunkwan University, Suwon, Republic of Korea; ^6^ Biomedical Institute for Convergence at SKKU (BICS), Sungkyunkwan University, Suwon, Republic of Korea

**Keywords:** contractility, induced pluripotent stem cells, organoid, cardiac models, IPSC-CM cardiomyocytes, particle image velocimetry

## Abstract

Induced pluripotent stem cell (iPSC)-derived cardiac organoids offer a versatile platform for personalized cardiac toxicity assessment, drug screening, disease modeling, and regenerative therapies. While previous image-based contractility analysis techniques allowed the assessment of contractility of two-dimensional cardiac models, they face limitations, including encountering high noise levels when applied to three-dimensional organoid models and requiring expensive equipment. Additionally, they offer fewer functional parameters compared to commercial software. To address these challenges, we developed an open-source, particle image velocimetry-based software (PIV-MyoMonitor) and demonstrated its capacity for accurate contractility analysis in both two- and three-dimensional cardiac models using standard lab equipment. Comparisons with four other open-source software programs highlighted the capability of PIV-MyoMonitor for more comprehensive quantitative analysis, providing 22 functional parameters and enhanced video outputs. We showcased its applicability in drug screening by characterizing the response of cardiac organoids to a known isotropic drug, isoprenaline. In sum, PIV-MyoMonitor enables reliable contractility assessment across various cardiac models without costly equipment or software. We believe this software will benefit a broader scientific community.

## Introduction

We developed an open-source particle image velocimetry-based software program for advanced contractility assessment of various cardiac models. PIV-MyoMonitor enables comprehensive quantitative assessment and advanced visualization of the contractile function across cardiac models, from single cells to 2D monolayers to 3D engineered heart tissues to 3D cardiac organoids. This novel tool can help investigate the biomechanics of cells and tissues for studying cardiovascular physiology and pathology.

Human induced pluripotent stem cell (hiPSC)-derived cardiac organoid is an attractive technology for drug discovery, disease modeling, and regenerative medicine. In particular, hiPSC-derived cardiac organoids can facilitate the prediction of drug-induced arrhythmia and cardiotoxicity in a patient-specific manner. To achieve this, it is essential to have a method to characterize contractile function of cardiac organoids in an accurate and reliable manner.

Several image-based contractility analysis methods have been developed to monitor cardiac contractile function such as edge detection (Harding et al., 1988; Steadman et al., 1988; Vescovo et al., 1989; He et al., 2003; Brito-Martins et al., 2008), optical flow/motion vector analysis (Hayakawa et al., 2012; 2014; Huebsch et al., 2015; Maddah et al., 2015; Pointon et al., 2017) and post-deflection tracking (Bremner et al., 2022). Edge detection method involves identifying the boundaries of cardiomyocytes and assessing how they change over time. The edge detection technique was more widely used in the early days, with specialized systems like IonOptix. This method has proven to be useful for some cardiac models such as mature rectangularly shaped single cardiomyocytes (Harding et al., 1988; Steadman et al., 1988; Vescovo et al., 1989). However, because it only measures the changes in the edges, it may not accurately predict the contraction in other cardiac models such as irregularly shaped immature hiPSC-derived cardiomyocytes or cardiac organoids. As an alternative, motion vector analysis, also known as optical flow analysis, has been more widely used these days. This method involves calculating displacement fields between consecutive images to predict the movement of cells. Motion vector analysis allows to quantify many parameters relevant to cardiac physiology such as beating frequency, maximum contraction/relaxation velocity, beating duration, time-to-decay, etc.

Several tools have been developed to enable measurements of cardiac contractility using motion vector analysis method that is open-source and accessible to non-specialist researchers (Huebsch et al., 2015; Sala et al., 2018; Grune et al., 2019; Scalzo et al., 2021). However, the existing tools still present a few limitations for improvement. First, most algorithms were validated using 2D cardiac models. In addition, most algorithms are fully automated or provide limited interactive options for data post-processing. This becomes important in analyzing 3D cardiac models especially when noise correction is needed. Furthermore, most reports utilize expensive apparatus such as high-speed cameras and microscopes. Additionally, most algorithms do not offer as comprehensive functional parameters and video outputs as those provided by commercially available and expensive software.

The aim of our study was to develop an open-source and accessible solution that enables multi-parameter analysis, provides interactive tools, and ensures adaptability for use with standard laboratory equipment. To enable utilization of standard laboratory equipment such as low-speed camera, we adopted a particle image velocimetry (PIV)-based algorithm to improve the motion tracking analysis. We demonstrate the PIV-based algorithm works well across variable speed camera (i.e. 10–150 frames per second, FPS). By benchmarking the gold-standard commercially available software, we describe an open-source software that provides comprehensive outcome analysis including 22 functional parameters and 3 different types of intuitive video outputs. In addition, we also included interactive functions for users to manually exclude noises that are not corresponding to real beating motion. We validated our PIV-based algorithm using multiple hiPSC-based cardiac models from single cells to monolayer models to engineered heart tissues to cardiac organoids. We demonstrated that our algorithm can predict drug response. We believe the cardiac research field would greatly benefit from the presented computational framework that provides a comprehensive and accessible solution for contractility measurement of cardiac models.

## Materials and methods

### PIV-based motion vector analysis and code availability

**FIGURE 1:**
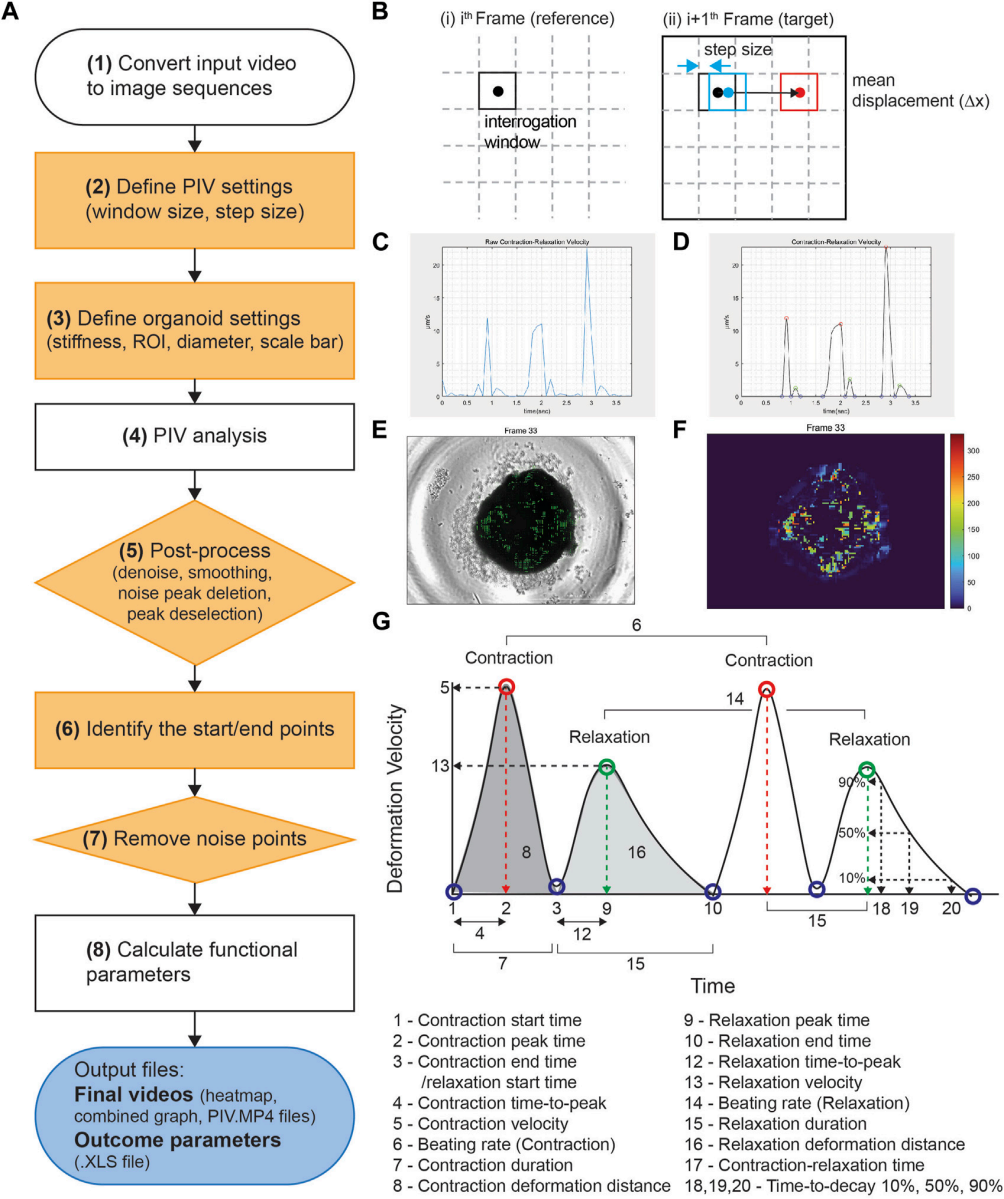
Workflow and schematics of PIV-MyoMonitor software program. **(A)** Workflow of the video processing procedure for an input cardiac beating video. Note that solid orange components indicate the user-interactive steps. **(B)** Schematic of the PIV algorithm. The algorithm calculates the displacement vector field between consecutive images based on the unique optical features within an interrogation window. **(C, D)** Time-displacement velocity graphs of a representative cardiac organoid **(C)** before and **(D)** after processing. Contraction peak (red), relaxation peak (green), Contraction/relaxation start and end points (blue). **(E)** An original cardiac organoid beating video overlayed with the displacement velocity vector field. **(F)** Heatmap visualization of the displacement velocity vector field at its maximum contraction. **(G)** Schematic of the functional parameters calculated by PIV-MyoMonitor.

PIV-MyoMonitor utilizes PIV algorithm (Thielicke and Sonntag, 2021) to enable more reliable and comprehensive contractility analysis of three-dimensional (3D) cardiac models. The workflow of PIV-MyoMonitor is outlined (Figure 1A). First, the contraction video of cardiac organoid is loaded and converted into image sequences. Then, user is asked to input appropriate PIV settings such as interrogation window size and step size, and organoid settings such as organoid stiffness and diameter. Next, PIV algorithm is used to calculate the mean deformation velocity of cardiac organoid. Briefly, the frames of the recorded contraction video of cardiac organoid are sub-divided into interrogation windows. An interrogation window from the *i*th frame is cross-correlated with a series of interrogation windows that were moved by the defined step size in the (i+1)th frame to find the maximally correlated one (Figure 1B). The maximum correlation between interrogation windows of consecutive frames is calculated using the Fast Fourier Transform (FFT) algorithm. The FFT algorithm converts an image from the spatial domain to the frequency domain. This transformation allows for the decomposition of an image by frequency, thereby facilitating a more accurate computation of correlation between windows. This process is done by the piv_FFTmulti function from PIVlab, an open-source toolbox in MATLAB. This function determines the position of maximally correlated window between consecutive images and subsequently calculate the displacement velocity vector. The calculated displacement vector field then undergoes post-processing including denoising, smoothing, noise peak deletion, and peak deselection (Figures 1C,D). In the workflow chart, the solid orange components indicate the steps that a user can provide inputs. The PIV-MyoMonitor code is available on GitHub at https://github.com/soahleelab/PIV-MyoMonitor.

### hiPSC maintenance and directed cardiac differentiation

We used previously established hiPSC lines, SCVI-111 (Matsa et al., 2016) and C2N3 (Jang et al., 2021) in this study. HiPSC maintenance and their directed cardiac differentiation were carried out as previously described (Buikema et al., 2020). Briefly, hiPSCs were maintained in DMEM/F12 (ThermoFisher, #11330057) supplemented with the essential eight (E8) growth factors in a Matrigel (Corning, #356231) coated (1:400 for 24 h) polystyrene 2D culture system. Upon 80%–90% confluency, cells were dissociated in PBS with 0.5 mM EDTA for 5–10 min at 37 C. Dissociation was performed with gentle trituration to obtain single cells of undifferentiated hiPSCs. Passaging was performed in 15,000 cells per cm^2^ to achieve low density and reach full confluency within 3–4 days. For the first 24 h after replating, 10 µM of ROCK inhibitor Y-27632 (Selleckchem, #S1049) was included in the hiPSC maintenance media. Once hiPSCs reach ∼80% confluency, the cells were passaged for maintenance or directed to differentiate into cardiomyocytes.

Cardiac differentiation of hiPSCs was performed using the previously described small-molecule-based canonical Wnt modulation protocol (Lian et al., 2013). Briefly, between day 0–2, hiPSCs were treated with CHIR99021 (Seleckchem, #S2924) concentrations (4.0, 5.0, 6.0 μM), in RPMI 1640-based differentiation media supplemented with B27 minus insulin (Gibco, #A1895601). Between day 2–4, Wnt-C59 (2.0 μM) (Selleckchem, #S7037) was added to the differentiation media. At day 4, the media was changed to the basal differentiation media (RPMI 1640) supplemented with B27 minus insulin. At day 6, B27 with insulin (Gibco, #17504-44) was added to the basal differentiation media. At day 8, to eliminate non-myocytes, RPMI 1640 minus glucose media supplemented with B27 plus insulin was used. At day 10, the media was replaced with the basal differentiation media supplemented with B27 plus insulin. At day 11, wells that contain more than 90% beating cells were treated with TrypLE Select Enzyme 10X (Gibco, #A1217702) at 37 °C for 10–40 min. Gentle rocking was performed every 10 min. Preparations of single dissociated cells were generated with very gentle trituration and transferred to a 15 mL conical tube containing a replating media (RPMI 1640 + B27 plus insulin +10% Knock Out Serum Replacement (Gibco, #10828028) + Thiazovivin 1.0 μM (Sigma, SML1045)). From day 12, the media were replaced with fresh cardiomyocyte (CM) maintenance media (RPMI + B27 plus insulin) every 2–3 days until day 30 unless specified otherwise for downstream applications.

### Single cell patterning of hiPSC-derived cardiomyocytes (hiPSC-CMs)

For single cell contractility assessment, day 27 hiPSC-CMs were plated on polyacrylamide hydrogels of 10 kPa stiffness with micro-patterned MatrigelTM to achieve physiological cardiomyocyte morphology exhibiting aspect ratio of 7:1 (Lee et al., 2020). After 3 days of culture on the patterned hydrogel, contractility of hiPSC-CMs were measured by recording contractile motion using high speed microscope camera (frame rate: 150 FPS, shutter speed: 1/150 s).

### Monolayers of hiPSC-CMs

For monolayer contractility measurement, the differentiated hiPSC-CMs were cultured in CM maintenance media until day 30, followed by recording of beating videos.

### Engineered heart tissues (EHTs)

EHTs were generated as described previously (Buikema et al., 2020). Briefly, 1 × 106 days 20 hiPSC-CMs were resuspended in EHT precursor solution containing bovine fibrinogen (F8630, 5 mg/mL), aprotinin (A1153, 2.5 μg/mL) and 10% Matrigel dissolved in CM replating media (RPMI 1640 + B27 plus insulin +10% knock-out serum +1 µM thiazovivin). Agarose-based EHT casting mold was created in 24-well plate using Teflon spacer (EHT technologies, Germany). 97 μL of cell-containing EHT precursor solution was mixed with 3 µL of thrombin (stock concentration 100 U/mL), and then quickly dispensed and casted into the casting mold with EHT PDMS posts (EHT technologies, Germany). The casted EHTs were incubated for 2 h for fibrin polymerization. The EHTs were transferred to CM maintenance media (RPMI 1640 + B27 plus insulin) supplemented with 33 μg/mL aprotinin for additional 10 days. Contractility measurement was performed at day 30.

### Cardiac organoid differentiation

**FIGURE 2:**
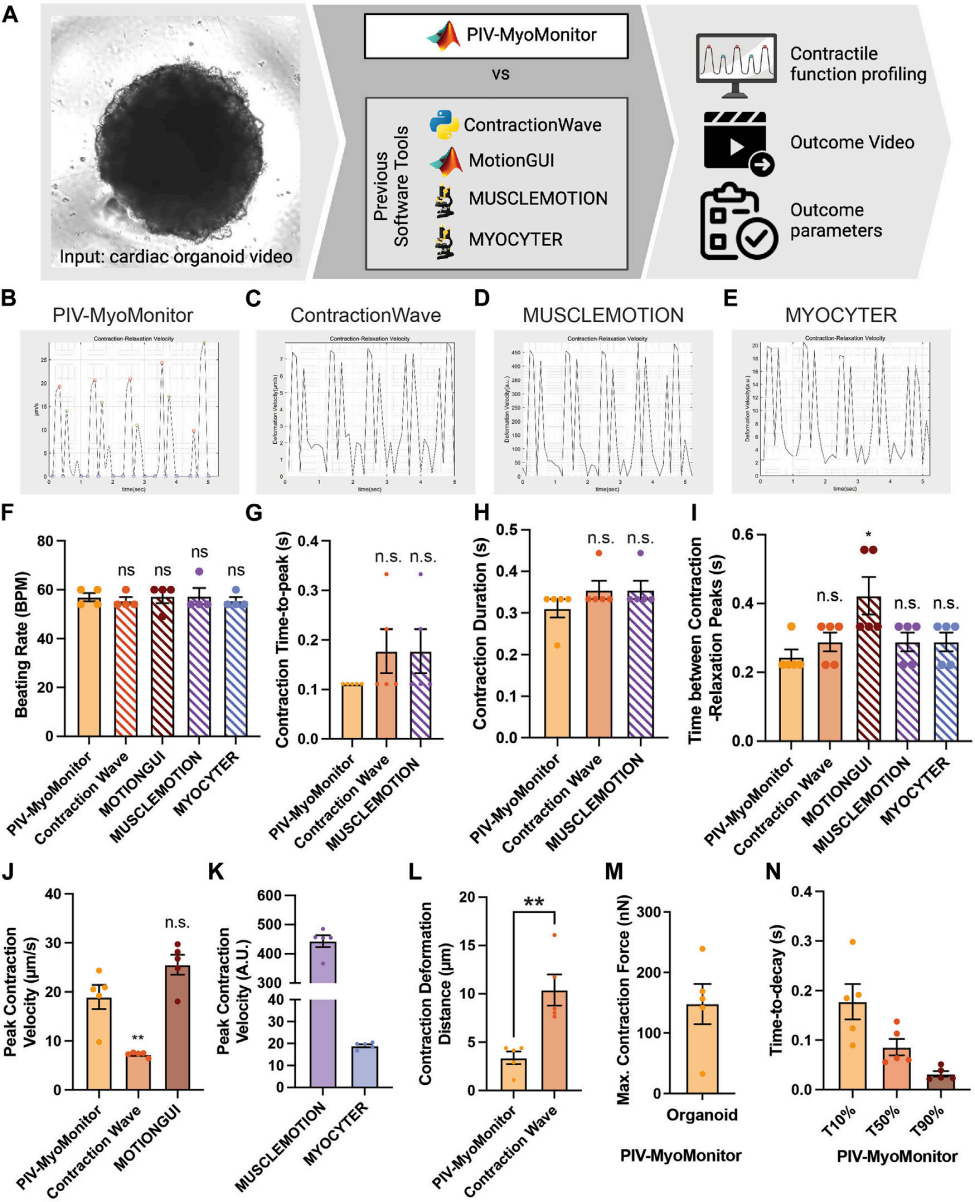
Comparison of PIV-MyoMonitor with published open-source contractility analysis software programs. **(A)** Schematic of the comparative study. **(B–E)** Time-displacement velocity graphs derived from using PIV-MyoMonitor, ContractionWave, MUSCLEMOTION, MYOCYTER. The graph from MotionGUI could not be reproduced because it did not export the raw data. **(F–N)** Beating rate, contraction time-to-peak, contraction duration, time between contraction-relaxation peaks, peak contraction velocity, contraction deformation distance, estimated maximum contractile force, and time-to-decay derived from using PIV-MyoMonitor, ContractionWave, MotionGUI, MUSCLEMOTION, and MYOCYTER. Data are presented as mean ± SEM. Statistical significance was determined by unpaired t-test. ns *p* > 0.05, *p < 0.05, **p < 0.01.

**FIGURE 3:**
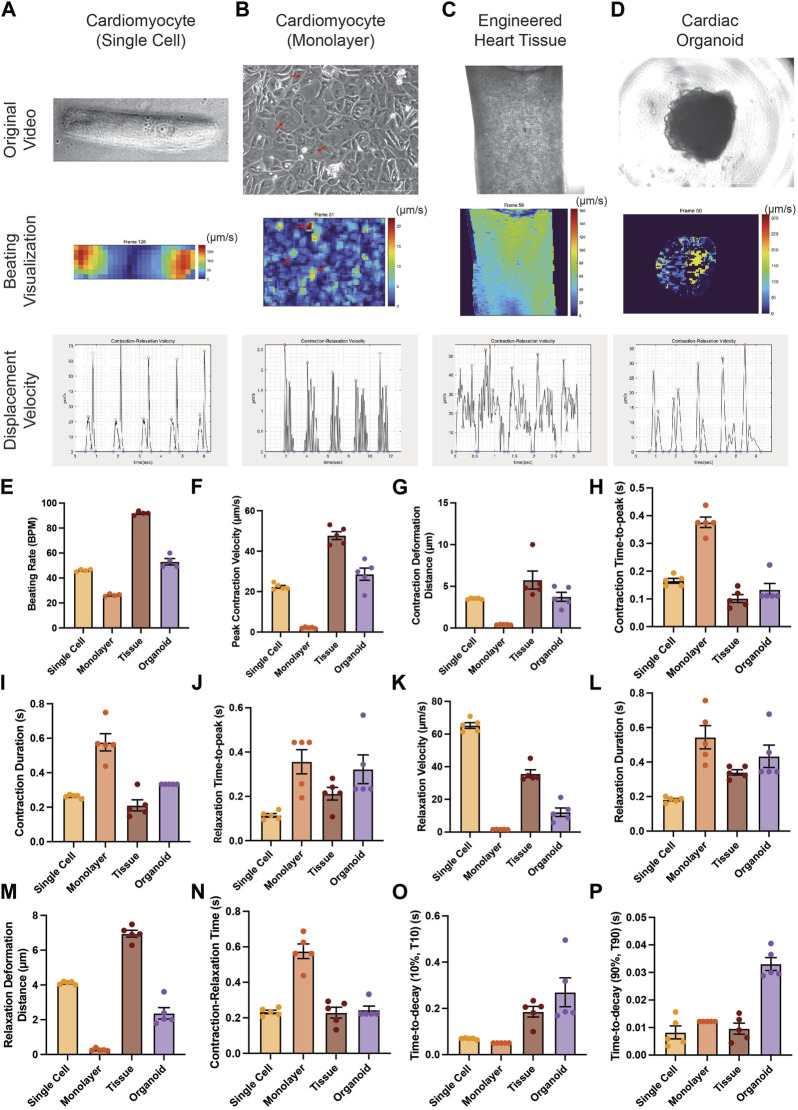
Applicability of PIV-MyoMonitor to various *in vitro* cardiac models. **(A–D)** Original images, displacement velocity heatmaps, and displacement velocity graphs of single-cell, monolayer, engineered heart tissue, and cardiac organoid models. **(E–P)** Functional parameters calculated using PIV-MyoMonitor. Data are presented as mean ± SEM.

hiPSC-based cardiac organoids were generated by modifying the protocol previously published (Lewis-Israeli et al., 2021). Briefly, hiPSC aggregates were formed by seeding hiPSCs at 10,000 cells/well on ultra-low attachment V-shaped 96-well plate (Sbio, MS-9096-VZ), previously rinsed with anti-adherence rinse solution (Stem Cell Technology, 07010). The plate was then centrifuged at 100 *g* for 3 min and placed in the CO2 incubator (day −2). After 24 h, 80 µL of media was changed to fresh Essential 8 medium. On day 0, media was changed to RPMI 1640 (Welgene, LM 011-01) based differentiation media supplemented with B27 minus insulin (Gibco, A1517001) containing 5 µM CHIR99021 (TOCRIS, 4,423) and incubated for 48 h. After 48 h (day 2), media was changed to RPMI-1640 + B27 minus insulin differentiation media containing 2 µM Wnt-C59 (Selleckchem, S7037) and incubated for 48 h. On day 4, the media was changed to fresh RPMI-1640 + B27 minus insulin differentiation media. From day 6 to day 30, the media was replenished with fresh RPMI-1640 + B27 with insulin (Gibco, 17504-44) differentiation media every other day. Spontaneously beating organoids start arising from day 9. Contractility measurement was performed at day 30. The organoids were generated from the C2N3 iPSC line (Figures 2, 3) and the SCVI-111 (Figure 5).

### Measurement of organoid stiffness using atomic force microscopy (AFM)

The stiffness of organoids was measured using AFM (XE-7; Park Systems, Suwon, Korea) with a Pyrex-Nitride Probe—Diving Board (PNP-DB) cantilever (resonance frequency: 17 kHz, force constant: 0.06 N/m, silicon nitride) from NanoWorld (Neuchâtel, Switzerland). The elastic modulus (E) was determined by quantitatively analyzing the Force-Distance (FD) curve. This involved modeling the contact region based on general contact mechanics theory (Chizhik et al., 1998). E values were calculated by fitting the data to the Hertz model for a pyramidal tip, using the XEI image processing program provided by Park Systems (Kontomaris and Malamou, 2020).

### Contractile force calculation

To estimate average contractile force generated by a cardiac organoid, we applied the theory of elasticity from solid mechanics. We assumed that an organoid is incompressible and purely elastic.

$$K=\frac{t{he\;change\;in\;pressure\;\left(-\Delta P\right)}^{*}}{the\;fractional\;change\;in\;volume\;\left(\frac{\Delta V}{V_{0}}\right)}$$

$$V_{0}:initial\;volume\;of\;organoid$$

ΔV:change in the volume of organoid

K:bulk modulus of organoid

*Negative pressure (– ΔP ) in the equation, is to describe the external force to compress an elastic material. In our organoid model system, it is the contractile stress generated by cardiomyocytes ( σ ) that leads to deforming the object (i.e., organoid).

Therefore,

$$\sigma =K\times \frac{\Delta V}{V}$$

$$\approx 3\times E\times \frac{\left(\frac{4\pi }{3}{\Delta r}^{3}\right)}{\left(\frac{4\pi }{3}{r_{0}}^{3}\right)}$$

$$where\;the\;tissue\;is\;assumed\;to\;be\;incompressible\;\left(poisson^{\prime }s\;ratio\;∼\;0.5\right)$$

F=σ×A

$$=3\times E\times \frac{{\Delta r}^{3}}{{r_{0}}^{3}}\times 4\pi {r_{0}}^{2}$$

σ:contractile stress generated by cardiomyocytes

F:contractile force generated by cardiomyocytes

A: surface area of organoid

E:elastic modulus of organoid

$$r_{0}:the\;radius\;of\;organoid$$

Δr:change in the radius of organoid . We used the displacement distance calculated from PIV analysis as Δr.

### Statistics

All analyses were performed using GraphPad software. All data was assumed to have a normal distribution. Statistical significance was evaluated with a standard unpaired Student’s t-test when stated otherwise (*p* < 0.05). For multiple-comparison analysis, one-way ANOVA with the Dunnett’s post-test correction was applied when appropriate (*p* < 0.05). All data are presented as mean ± s.e.m.

## Results

### PIV-MyoMonitor: PIV algorithm enables robust contractility analysis of cardiac organoids

We developed PIV-MyoMonitor to enable more reliable and comprehensive contractility analysis of 3D cardiac models. PIV algorithm is used for calculating the mean deformation velocity of cardiac organoid during contraction and relaxation. For PIV calculation, user is asked to input appropriate PIV settings such as interrogation window size and step size, which needs to be empirically determined. As setting an appropriate interrogation window size and step size is key for reliable prediction of PIV-based contractility analysis, we examined the effect of varying interrogation window sizes (8, 16, 32, 64 pixels) and step sizes (4, 8, 16 pixels) on the calculated displacement velocity vector field. We chose to evaluate the frame when the cardiac organoid is contracting the most (Frame 33, time = 2.9 s) (Supplementary Figure S1). We found that when interrogation window size is 64 pixels, the contraction velocity field was discontinuous as evident by abrupt velocity difference between adjacent interrogation windows (Supplementary Figure S1. A–C, white arrow). Setting the interrogation window to be 32 pixels or below showed the velocity field to be more continuous (Supplementary Figure S1. D–I). On the other hand, reducing the step size from 16 pixels to 4 pixels resulted in a denser velocity vector field information at the expense of increased computation time. Considering these factors, we determined the optimal interrogation window size to be 32 pixels and the step size to be 8 pixels for the further cardiac organoid contractility analysis.

After PIV calculation, the calculated raw displacement vector field undergoes post-processing including denoising, smoothing, noise peak deletion, and peak deselection (Figures 1C,D). We made this post-processing procedure to be user interactive. User can set the noise threshold, remove the noise peaks, as well as confirm or deselect the start/peak/end points that are automatically selected. Finally, the analysis outputs multiple informative videos including an organoid beating video overlaid with velocity vector field, heatmap, and time-velocity graph temporally matched with organoid beating video (Figures 1F,G, Supplementary Video S1–S3). Twenty-two functional parameters are exported, including beating rate, peak deformation velocity during contraction and relaxation, as well as contraction/relaxation deformation distance (Figure 1H).

### PIV-MyoMonitor provides more comprehensive quantitative analysis and advanced visualization compared to other open-source contractility evaluation software programs.

Several open-source contractility evaluation software programs, including ContractionWave, MotionGUI, MUSCLEMOTION, and MYOCYTER, are available (Huebsch et al., 2015; Sala et al., 2018; Grune et al., 2019; Scalzo et al., 2021). To compare the analysis capacity of PIV-MyoMonitor to others, we used a cardiac organoid video for contractile function profiling and subsequently compared the output parameters and videos (Figure 2, Supplementary Table S1). We found that the contraction-relaxation graph was generally similar in shape such as the number of peaks and peak-to-peak distances (Figures 2B–E). The graph from MotionGUI is not shown because not all the raw datapoints were exported for replotting. For quantitative comparison, we plotted and performed statistical analysis on parameters reported by five software programs. Each data point represents information associated with individual beating peaks of an organoid (Figures 2F–N). Multiple data points showed identical values (Figures 2F–N), indicating consistent beating behavior throughout the recording period. Time-relevant variables such as beating rate, contraction time-to-peak, contraction duration evaluated from PIV-MyoMonitor were not statistically different from those from the other programs (Figures 2F–I). This indicates all five programs successfully analyzed the timing of contraction and relaxation movements. One thing to note is that some parameters, such as contraction duration and time between contraction-relaxation peaks, were not directly reported by the other programs. Furthermore, even reported value, such as beating rate, needed to be re-calculated due to the presence of noise peaks (Figures 2F–I, manually calculated data are indicated by hatched bar). Peak contraction velocity was estimated to be comparable between PIV-MyoMonitor (18.96 ± 1.721 μm/s) and MOTIONGUI (25.51 ± 2.027 μm/s), while it was statistically lower when using ContractionWave (7.258 ± 0.2103 μm/s) (Figure 2J). Peak contraction velocity calculated from MUSCLEMOTION and MYOCYTER were not directly comparable because it was reported in an arbitrary unit (Figure 2K). Contraction deformation distance, an important parameter positively correlated with contractile work, was calculated only by PIV-MyoMonitor and ContractionWave (Figure 2L). PIV-MyoMonitor is the only software program that automatically calculates maximum contractile force and time-to-decay (T10%, T50%, T90%) (Figure 2M,N). In summary, PIV-MyoMonitor algorithm enables comprehensive profiling of contractile behavior of cardiac organoids by reporting 22 quantitative functional parameters that are not available by other open-source software programs.

Furthermore, PIV-MyoMonitor enables advanced visualization of organoid contraction. PIV-MyoMonitor exports 4 videos: 1) rawCombine video is for identifying real contraction/relaxation signals and denoising process (Supplementary Video S4), 2) PIV-processed video is for visualizing deformation velocity vector fields (Supplementary Video S5), 3) heatmap_processed video is for visualizing the intensity of deformation velocity (Supplementary Video S6), and 4) combined final video is the finalized annotated contraction-relaxation plot with the original beating video (Supplementary Video S7). Among the four programs, MYOCYTER is the only program that provides a video output displaying the organoid beating along with contraction-relaxation plot, even though the video frame rate is changed from the original video (Supplementary Video S8). ContractionWave exports an image sequence of a heatmap that can be encoded into a heatmap video. In summary, PIV-MyoMonitor provides diversely processed videos for advanced visualization of cardiac contractile behavior.

### PIV-MyoMonitor can be applied to various *in vitro* cardiac models

We examined whether PIV-MyoMonitor can be used for analyzing contractility of various *in vitro* cardiac models from single cell to monolayer to tissue to organoid (Figure 3). PIV-MyoMonitor successfully detected single peaks for contraction (red) and relaxation (green) in a single-cell-patterned hiPSC-CM model (Figure 3A). In agreement with previous reports (Ribeiro et al., 2015), PIV-MyoMonitor predicted that contraction and relaxation of a single-cell-patterned hiPSC-CM were maximal on the edge of the cell (Figure 3A, Supplementary Video S9,S10). Monolayer hiPSC-CMs showed unsynchronized contraction (Supplementary Video S11), which was reflected by multiple contraction and relaxation peaks during one cycle (Figure 3B). Heatmap visualization revealed a set of iPSC-CMs that contracted stronger than other cells (Figure 3B, red arrows). EHT also exhibited multiple peaks in contraction and relaxation, indicating the wave-like motion associated with contraction (Figure 3C, Supplementary Video S12). Heatmap video of the EHT model showed that contraction propagates diagonally from top right to bottom left (Figure 3C, Supplementary Video S13). Cardiac organoid model showed single contraction and relaxation peaks, indicating relatively synchronized contractile behavior (Figure 3D, Supplementary Video S14). We showed that PIV-MyoMonitor can quantitatively examine functional parameters across various *in vitro* cardiac models (Figure 3E–P). In summary, PIV-MyoMonitor can successfully be applied for quantitative analysis of various *in vitro* cardiac models and their visualization.

### PIV-MyoMonitor can analyze contraction videos with various frame rates

**FIGURE 4:**
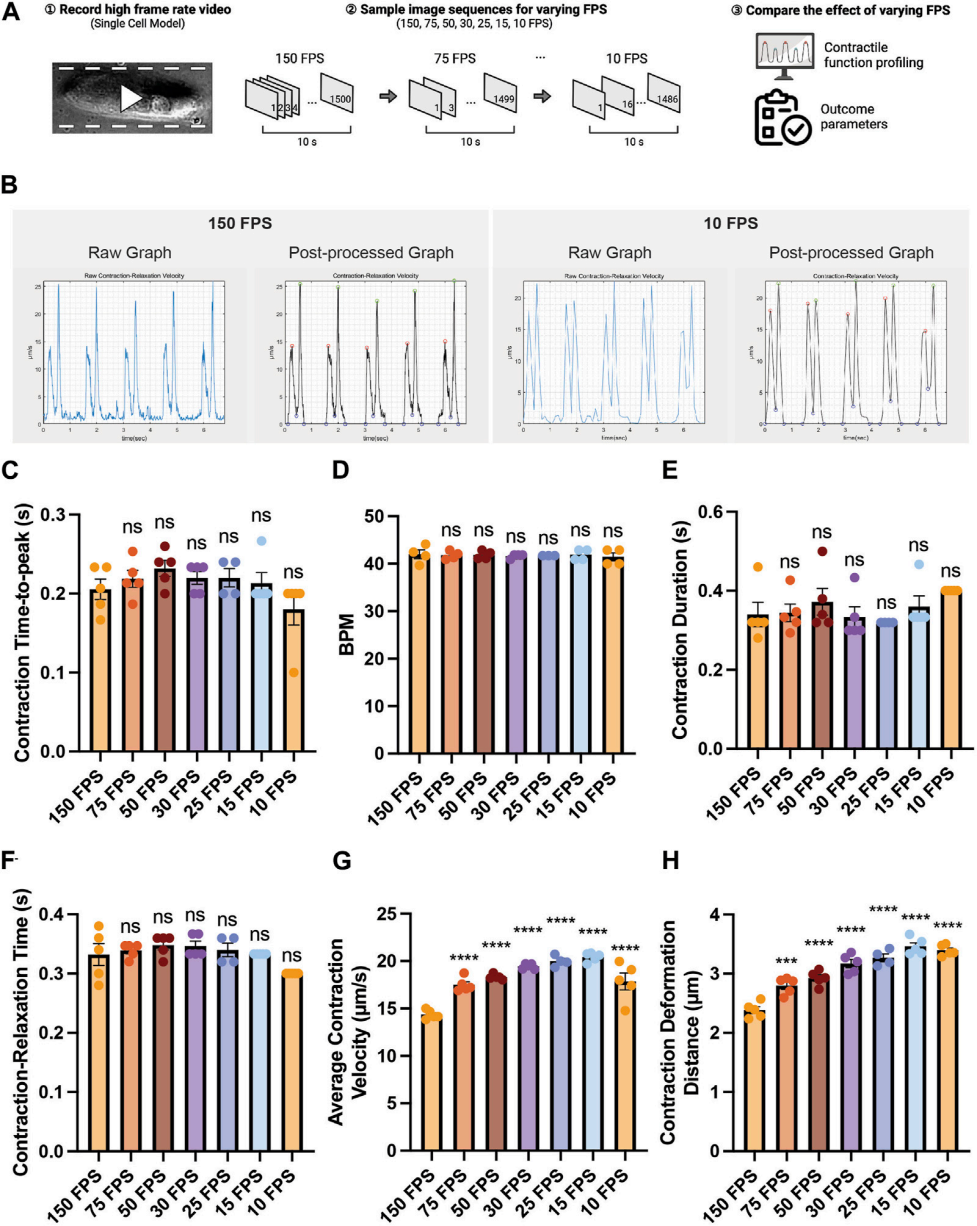
Effect of the video frame rate on contractility analysis. **(A)** Schematic of the study design. **(B)** Raw and post-processed time-displacement velocity graphs analyzed from representative videos (150 FPS and 10 FPS). **(C–H)** Effect of decreasing video frame rate on **(C–F)** time-related functional parameters and **(G, H)** length-related functional parameters. Data are presented as mean ± SEM. Statistical significance was determined by a one-way ANOVA with Dunnett’s multiple comparisons test (control: 150 FPS). ns *p* > 0.05, ****p < 0.0001.

Since some laboratories are not equipped with high-speed cameras, we sought to examine the effect of varying the frame rates of an input beating video on contractility outcome analysis. A contraction video of a single hiPSC-CM was recorded using a high-speed camera (150 FPS), and the captured image sequences were sampled to generate videos with lower frame rates (75, 50, 30, 25, 15, 10 FPS) (Figure 4A). We found that varying frame rate did not lead to a substantial change in contraction-relaxation velocity graph pattern (Figure 4B). The graph from the 10 FPS video was smoother than that from 150 FPS due to less datapoints. We found that varying frame rate did not significantly affect the time-related parameters such as contraction time-to-peak, beating rate (beats per minute, BPM), contraction duration, contraction-relaxation time (*p* > 0.05), indicating that videos with as low as 10 FPS frame rate can be used to successfully examine these parameters (Figures 4C–F). Average contraction velocity and contraction deformation distance were found to increase with decreasing frame rate (Figures 4G,H). For example, average contraction velocity of 10–75 FPS videos were significantly larger than that of 150 FPS video (*p* < 0.0001). Contraction deformation distance gradually increased from 2.38 μm to 3.39 μm with decreasing FPS.

### PIV-MyoMonitor can examine drug responses in cardiac organoid in a longitudinal and non-destructive manner

**FIGURE 5:**
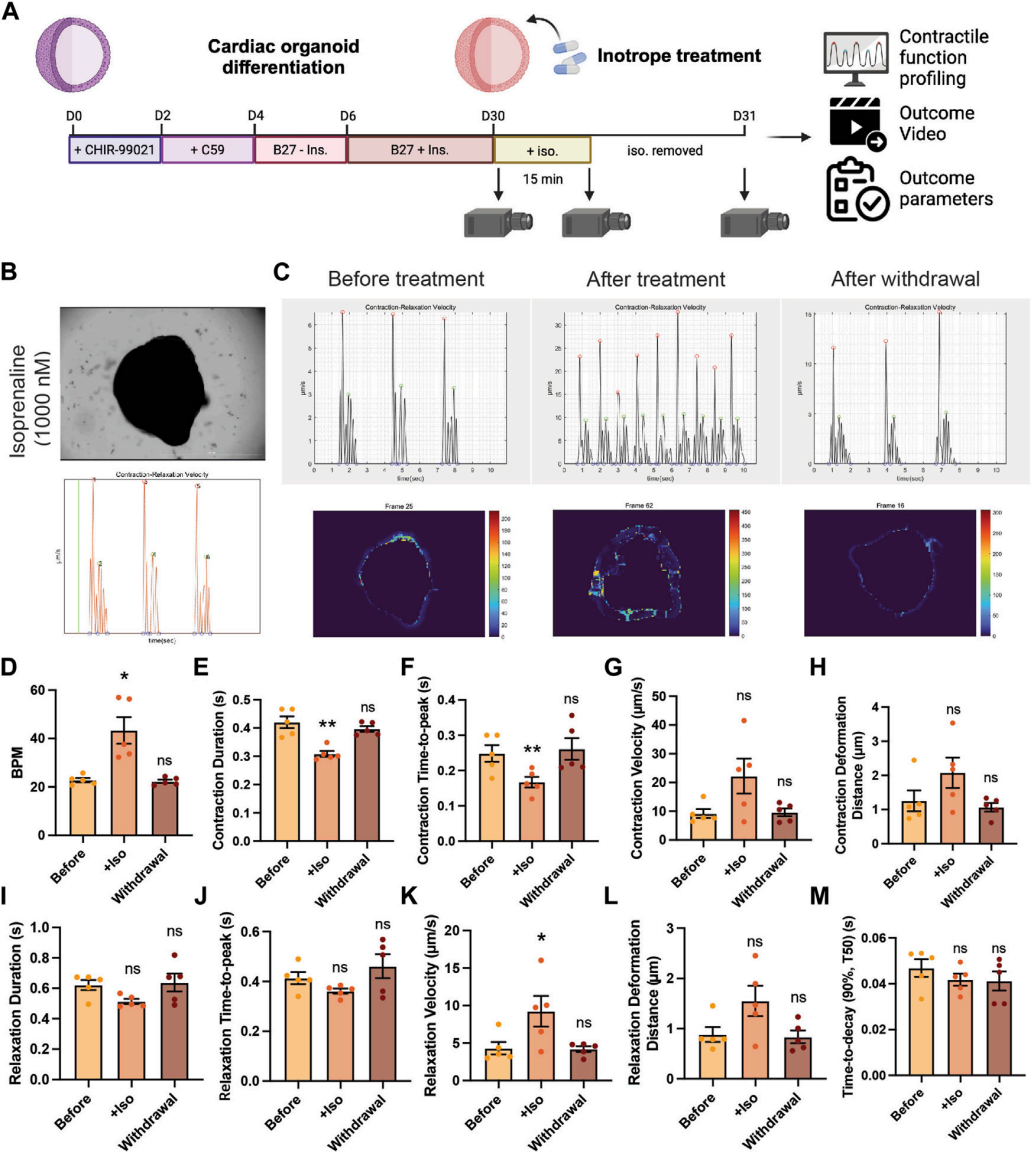
Validation of using PIV-MyoMonitor in contractility analysis of cardiac organoid in response to isoprenaline treatment. **(A)** Schematic of the experimental design. **(B)** A snapshot of the final video of the original beating video along with a time-displacement velocity graph. The green bar helps evaluate whether the peaks correspond to actual beating behaviors. Please refer to Supplementary Video S13. **(C–M)** Time-displacement velocity graphs, heatmap images, and selected functional parameters of a representative cardiac organoid before and after isoprenaline treatment as well as after its withdrawal. Data are presented as mean ± SEM. Statistical significance was determined by paired t-test. ns *p* > 0.05, *p < 0.05, **p < 0.01.

Next, we examined the feasibility of using PIV-MyoMonitor for evaluating contractility of cardiac organoids in response to various cardiac drugs. We selected a widely used inotropic agent for this evaluation, isoprenaline. Isoprenaline is a β-adrenergic receptor agonist that exerts positive chronotropic and inotropic effects. Beating videos of day 30 cardiac organoids were recorded before and after 15-min treatment with isoprenaline as well as 1 day after withdrawal of the drug (Figure 5A). Beating videos of cardiac organoids were analysed using PIV-MyoMonitor. The output video was used to identify contraction and relaxation peaks that corresponds to actual contractile and relaxation behaviours (Figure 5B, Supplementary Video S15). Before the treatment, day 30 cardiac organoids showed the average beating rate of 24.3 ± 3.22 BPM, which significantly increased after treatment of 1 µM isoprenaline (Figures 5C,D). In agreement, average contraction duration decreased from 0.42 s to 0.31 s, and contraction time-to-peak also shortened from 0.24 s to 0.17 s in response to isoprenaline treatment (Figures 5E,F). Average contraction velocity and contraction deformation distance were 9.18 μm/s and 1.26 µm, respectively at baseline (Figures 5G,H). Isoprenaline treatment led to 2.42- and 1.65-fold increase in contraction velocity and contraction deformation on average despite no statistical significance due to high organoid-to-organoid variability. After withdrawal of isoprenaline, the contraction kinetics and deformation distance returned back to the baseline (Figures 5D–H). The changes in relaxation kinetic (i.e., relaxation duration, relaxation time-to-peak, relaxation velocity) and relaxation deformation distance in response to isoprenaline treatment and withdrawal followed the similar trend to the contraction (Figures 5I–L). Relaxation time-to-decay was unaffected by isoprenaline treatment (Figure 5M).

In summary, we demonstrated that PIV-MyoMonitor can be used to profile the contractile function of cardiac organoids longitudinally and non-destructively in response to isoprenaline. The analysis showed the expected positive inotropic and chronotropic effects of the drug. This result supports that PIV-MyoMonitor is a useful tool for analyzing short- and long-term drug effects on 3D cardiac organoids.

## Discussion

### Result highlights and conclusion

We developed a novel contractility analysis software based on PIV algorithm (Figure 1). Traditionally, optical-flow-based methods have been widely used for analyzing the contractility of various cardiac models. However, these methods present several limitations when applied to 3D tissue models. Optical flow is a method used in computer vision to estimate the motion of objects or features in image sequences. It assumes that the intensity patterns in an image change smoothly over time. Therefore, the accuracy of analysis may be compromised in situations involving abrupt changes in the shape of objects and the pixel intensity comprising the object. Additionally, it can struggle to accurately track the movement of objects in videos when they overlap and are hard to be distinguished from the background (McIlvenny et al., 2023). Consequently, applying optical-flow-based method may pose challenges in analyzing 3D tissue models due to the deformation of cell shapes during contraction and the overlapping of cells. PIV algorithm calculates the movement of objects or features based on the pattern similarity within interrogation windows. PIV is a technique often used in fluid dynamics and it requires embedding of small fluorescent particles in fluid to introduce “pattern”. In biological system, subcellular features or cell distribution pattern serves as a unique identifiable pattern that can be used to track displacement. Furthermore, transformation of image pattern to the frequency domain using FFT enables more accurate calculation for finding maximally correlated interrogation windows between consecutive images. This enables precise analysis of dynamic processes such as contraction and relaxation in 3D cardiac organoid models. In sum, PIV-MyoMonitor overcomes the limitations of optical-flow-based methods and demonstrates its robust capability in analyzing the contractility of various *in vitro* cardiac models.

For successful PIV analysis, it is necessary to optimize the window size and step size for each model, while considering computational load. We demonstrated the optimization procedure of PIV settings for organoid model (Supplementary Figure S1). In general, increasing window size improves the specificity of interrogation window, while decreasing step size leads to more thorough cross-comparison of interrogation windows. In the case of our cardiac organoid model, we found that utilizing a window size of 32 pixels and a step size of 8 pixels led to accurate quantification of beating behavior while maintaining a reasonable computation time.

PIV-MyoMonitor offers several advantages over the four other available programs. First, PIV-MyoMonitor provides more accurate contractility analysis of cardiac organoids recorded using cameras with low frame rate (Figure 4, Supplementary Figure S4). We demonstrated that the deviation in contraction velocity between the reduced 10 FPS video and the original 150 FPS video was the smallest (i.e. 1.24-fold increase) when using PIV-MyoMonitor, compared to the other programs (MotionGUI: 1.84-fold increase; Contraction Wave: 0.45-fold decrease; MYOCYTER: 3.62-fold increase; MUSCLEMOTION: 5.74-fold increase). Moreover, PIV-MyoMonitor provides additional functional parameters that are not calculated by the other programs, resulting in a total of 22 functional parameters reported. For example, contraction deformation distance is an indirect parameter for contractile work, time-to-decay during relaxation is a parameter related to Ca2+ recycling, and contraction time-to-peak reflects the rate of depolarization (Figure 2). These additional functional parameters can help identify potential molecular and cellular mechanisms affected by genetic and pharmacological perturbations. Furthermore, advanced visualization of cardiac contraction provided by PIV-MyoMonitor offers great advantage over other programs (Supplementary Video S4–S8). A final video output that combines original beating video with raw analysis plot side-by-side (annotated as “combined.mp4”) enables more accurate data post-processing (Supplementary Video S7). For example, users can choose to exclude noise peaks and adjust the start, end, and peak timepoints of contraction and relaxation to annotate the beating behavior more accurately. Heatmap and motion vector videos are particularly helpful in providing spatial information about the direction and intensity of organoid beating behaviors. Lastly, PIV-MyoMonitor offers user-interactive post-processing functions. While automatic post-processing may be more beneficial for high-throughput analysis, user-interactive functions can be particularly useful when analyzing 3D organoids where noise peaks cannot be automatically distinguished from true peaks. We believe PIV-MyoMonitor serves as an advanced solution for analyzing the contractility of 3D cardiac organoids, contributing to the broader cardiac community.

### Limitations of study

While PIV-MyoMonitor presents considerable strengths in evaluating the contractility of cardiac organoids, certain limitations warrant attention. One of the primary limitations observed in our study is the limited ability of our software in evaluating thick and dark tissue samples. This is not necessarily due to the algorithm; rather it is due to the optical limit. As the tissue becomes thicker and denser, light cannot penetrate it as much, leading to similar dark patterns in the tissue area. This similarity in patterns results in inaccurate correlation of interrogation windows, and consequently, inaccurate prediction of displacement. We observed many noise peaks that did not correspond to actual beating behaviors particularly in the dark, core region of cardiac organoid (Supplementary Figure S2A, red: real peaks, the rest: noise peaks). To exclude noises resulting from the dark portion of the organoids, the brightness and contrast of contraction videos can be adjusted (Supplementary Figure S2B). Adjusting the brightness and contrast to make the underexposed area completely dark (i.e., pixel intensity = 0) can reduce inaccurate correlation and hence minimize the production of noise peaks. Alternatively, masking the dark region can also resolve the issue with noise. Since noise predominantly emerges from the core of an organoid, masking out the core region can aid in noise elimination. The final strategy involves introducing identifiable features into cardiac organoids, such as using DAPI staining or fluorescent protein labeling. The introduction of new fluorescent features improves pattern specificity in the core region, facilitating pattern correlation even in the dark tissue portion where identifiable features were absent using brightfield imaging. Lastly, we acknowledge the limitations regarding assumptions we made for contractile force estimation. Cardiac organoids are viscoelastic. Also, they are not perfectly incompressible although biological tissues are often considered nearly incompressible (Chagnon et al., 2017). While their compressibility can become more apparent when subject to high levels of deformation, incompressibility is often a good assumption to make in the range of deformation that organoid is undergoing.

## Data Availability

The raw data supporting the conclusion of this article will be made available by the authors, without undue reservation.

## Supplementary material

The Supplementary for this article can be found online at: https://www.frontiersin.org/articles/10.3389/fbioe.2024.1367141/full#supplementary-material

SUPPLEMENTARY TABLE S1 Comparison between PIV-MyoMonitor and four other open-source software programs.

SUPPLEMENTARY FIGURE S1 Optimization of PIV setting for contractility analysis of cardiac organoids. **(A–I)** Examination of interrogation window sizes ranging from 8 to 64 pixels and step sizes from 4 to 16 pixels.

SUPPLEMENTARY FIGURE S2 Proposed strategies for reducing noise production in the center region of cardiac organoids. **(A)** Original cardiac organoid image and the representative displacement velocity maps in vector and heatmap presentation. Red arrows indicate the peaks corresponding to the real beating motion. **(B)** Effect of brightness and contrast **(B/C)** adjustment. **(C)** Effect of masking.

SUPPLEMENTARY FIGURE S3 Stiffness measurement of a cardiac organoid using atomic force microscopy. Data are presented as mean ± SEM.

SUPPLEMENTARY FIGURE S4 Evaluation of the analysis capacity of reduced FPS videos using four open-source contractility software programs. The effect of decreasing video frame rates on calculating average contraction velocity by **(A)** Contraction Wave, **(B)** MOTIONGUI, **(C)** MUSCLEMOTION, **(D)** MYOCYTER. Data are presented as mean ± SEM. Statistical significance was determined by unpaired t test. ns p>0.05, * p<0.05, ** p<0.01, *** p<0.001, **** p<0.0001.
