# Supplementary material for: PIV-MyoMonitor: an accessible particle image velocimetry-based software tool for advanced contractility assessment of cardiac organoids
Source: Front Bioeng Biotechnol. 2024 Mar 12;12:1367141. doi: 10.3389/fbioe.2024.1367141 (PMC10964252; doi:10.3389/fbioe.2024.1367141)
Supplement: Supplementary file 3 [file Table1.DOCX]

Supplemental Table 1. Comparison between PIV-MyoMonitor and four other open-source software programs

| **Category** | | **PIV-MyoMonitor** | **MotionGUI** | **Contraction Wave** | **MUSCLEMOTION** | **MYOCYTER** |
| --- | --- | --- | --- | --- | --- | --- |
| Set-up | Development Environments | MATLAB | MATLAB | Python | ImageJ | ImageJ |
|  | User Interface | Interface based on console window and figure window | GUI | GUI | ImageJ-based interfaces | ImageJ-based interfaces |
|  | Input Format | Video (all the formats supported by MATLAB including AVI, MP4) | Image sequence | Video, image sequence | Image sequence | Video (uncompressed  AVI only) |
| Pre-processing | ROI Selection | O | O | X | X | O |
|  | Edge Detection | O | O | O | O | O |
| Processing | Displacement  Calculation Method | Maximum correlation of interrogation window using FFT | Block matching  using macro block | Dense optical flow based on Farneback algorithm | Subtraction of pixel intensity between  two consecutive frames (for speed calculation) or reference frame and the frame of interest (for amplitude calculation) | |
| Post-processing | Denoise Method | (1) using threshold,  (2) manual deletion | X | Using threshold | Applying a Gaussian filter (blur) | Dynamic thresholding |
|  | Peak Selection | (1) using threshold,  (2) manual selection | Using threshold | Using threshold | X (automatic thresholding) | X (automatic thresholding) |
|  | Start-end Point Selection | Selection of candidates based on polynomial followed by manual selection | X | (1) using threshold,  (2) manual selection is possible but complex | X | X |
| Data Export | Displacement Velocity/Speed Raw Data Points | O | X (peak  data only) | O | O | O |
|  | BPM | O | O | △ (manual calculation needed) | △ (manual calculation needed) | △ (manual calculation needed) |
|  | Time-to-decay | O | X | X | X | X (only for amplitude) |
|  | Contraction/Relaxation Duration | O | X | O | O | X (only for amplitude) |
|  | Time-to-peak | O | X | O | O | X (only for amplitude) |
|  | Estimated Contraction Force | O | X | X | X | X |
|  | Max Velocity (length unit) | O (μm/s) | O (μm/s) | O (μm/s) | △ (a.u.) | △ (a.u.) |
|  | Deformation Distance /Amplitude (length unit) | O | X | O | △ (a.u.) | △ (a.u.) |
| Visualization  Output | Graph Output | O | X | X | O | O |
|  | Combined Video Output  (graph + beating video) | O | X | X | X | △ (FPS altered) |
|  | Heatmap Visualization | O | X | △ (image sequence) | X | X |
|  | Visualization of Motion Vector/Optical Flow | O | O | △ (image sequence) | X | X |
